# Lipid Rafts and Redox Regulation of Cellular Signaling in Cholesterol Induced Atherosclerosis

**DOI:** 10.2174/157340310793566181

**Published:** 2010-11

**Authors:** Betul Catalgol, Nesrin Kartal Ozer

**Affiliations:** Department of Biochemistry, Faculty of Medicine, Marmara University, 34668 Haydarpasa, Istanbul, Turkey

**Keywords:** Atherosclerosis, hypercholesterolemia, lipid rafts, reactive species, redox regulation, signaling.

## Abstract

Redox mediated signaling mechanisms play crucial roles in the pathogenesis of several cardiovascular diseases. Atherosclerosis is one of the most important disorders induced mainly by hypercholesterolemia. Oxidation products and related signaling mechanisms are found within the characteristic biomarkers of atherosclerosis. Several studies have shown that redox signaling via lipid rafts play a significant role in the regulation of pathogenesis of many diseases including atherosclerosis. This review attempts to summarize redox signaling and lipid rafts in hypercholesterolemia induced atherosclerosis.

## INTRODUCTION

Atherosclerosis, a chronic inflammatory disease which is characterized by the accumulation of plasma lipoproteins that carry cholesterol and triglycerides in the arteries, is one of the major causes of morbidity and mortality worldwide. This accumulation results in the proliferation of certain cell types within the arterial wall. Arterial wall consists of three layers with different cell types and extracellular matrix. The outermost layer, tunica adventitia, includes fibroblasts, type I collagen fibers, elastic network, lymphatic and tiny blood vessels. The middle layer, tunica media, is circularly arranged with smooth muscle cells and in between the smooth muscle layers, elastic network, collagen and proteoglycans take place. The innermost layer, tunica intima, includes single layer endothelial cell lines in the luminal arterial surface. These cells attach on a basement membrane of extracellular matrix and proteoglycans that is bordered by the internal elastic membrane. Endothelial cells form a physical and functional barrier between flowing blood and the stroma of the arterial wall and regulate a wide array of processes including thrombosis, vascular tone, and leukocyte trafficking among others [1].

In the atherosclerotic process, macrophage foam cells are formed with the rapid transformation of phagocytic monocytes penetrated into the subendothelial space and atherogenic lipoproteins like modified low density lipoprotein (LDL) are uptaken by receptor-mediated endocytosis mechanism [2,3]. Following the endocytosis, these cells have an appearance loaded with lipid droplets rich in cholesteryl esters [4]. These foam cells also known as ‘fatty streaks’ and adaptive thickening of the intima are accepted as the main visible lesions at the early stage of the pathogenesis [1,4]. Endothelial dysfunction has been proposed as long-term atherosclerotic lesions which initiates the inflammatory mechanisms and is used as an important diagnostic and prognostic factor [5, 6]. Several biological processes classified as the reasons for advanced lesions in atherosclerosis such as proliferation of intimal smooth muscle cells, accumulation of extracellular matrix components such as collagen, elastic fibers and proteoglycan, and cholesteryl ester and free cholesterol accumulation within the cells and in the surrounding connective tissues [7]. During the progress of the disease, it takes time for the disruption of an atherosclerotic lesion and leading to thrombosis and decrease in oxygen supply to target organs such as heart and brain. As a result of these reduced blood flow, heart attack and stroke are occured referred to as coronary artery disease and cerebrovascular disease [1].

Enzymatically hydrolyzed LDL (E-LDL) [8], oxidized LDL (ox-LDL) [9] and modified LDL by advanced glycation end products (AGEs) are the types of lipids taken up by macrophages. The term E-LDL is used for proteolytically cleaved apoB and hydrolysed core cholesteryl esters, leading to liposome-like particles present at early stages in atherosclerotic lesions [8, 10]. With LDL oxidation, denatured apoB molecule shows an increase in the platelet-activating factor (PAF)-acetylhydrolase-like activity with a PLA2-like activity that strips phosphatidylcholine from the ox-LDL surface [11, 12]. Following this, ox-LDL particles aggregate and form polar surface with the remaining phospholipids on the aggregated particles. AGEs, those may cause LDL modification, are formed by nonenzymatic glycation reaction between a reducing sugar and a free amino group on a protein, lipid, or nucleic acid [13].

Cellular uptake of these atherogenic lipids and lipoproteins are mediated by several receptors as summarized in Table **1**. LDL receptors are generally shown to be downregulated during cholesterol uptake. Scavenger receptors (SRs) are the most abundant receptors expressed on macrophages and foam cells in atherosclerotic lesions [3]. CD36 takes the most important place in the scavenger receptors playing role in atherosclerotic process. CD36 is a raft associated glycosylated protein with an 88 kDa molecular weight. Studies showed that CD36 is palmitoylated in cysteine residues of N- and C-terminal of both cytosolic tails and this structure plays important role for the internalization of CD36 in caveloae and lipid membranes. Various ligands such as ox-LDL, apoptotic cells, AGEs bind to the region localized between 155-183 amino acids in the structure of scavenger receptor cluster of differentiation 36 (CD36) [1].

Hyperlipidemia and other cardiovascular risk factors containing age, obesity, hypertension, diabetes mellitus and serum cholesterol are connected with the development and progression of atherosclerotic lesions, plaque rupture, and vascular thrombosis. As an autosomal dominant disorder, familial hypercholesterolemia affects every 1 person in 500 from the general population [1, 3].

## OXIDATION PRODUCTS AND REDOX SIGNALING MECHANISMS IN ATHEROSCLEROSIS

Free radicals contain unpaired electrons in their outer orbitals and take place in oxidation reactions easily. Free radicals include reactive species (RS) such as reactive oxygen species (ROS) and reactive nitrogen species (RNS). RS damage cellular components such as proteins, lipids, carbohydrates, and nucleic acids [14]. Following the interactions of RS with cellular components, several products are known to be produced. The main investigated products are malondialdehyde (MDA) and 4-hydroxynonenal (HNE) for lipid peroxidation, 8- hydroxydeoxyguanosine for DNA oxidation and protein carbonyls, nitrotyrosines for protein oxidation [15, 16]. In this direction, the term redox signaling defines a process wherein free radicals (particularly reactive oxygen species) and other related species act as messengers in biological systems [17].

**Fig. (1):**
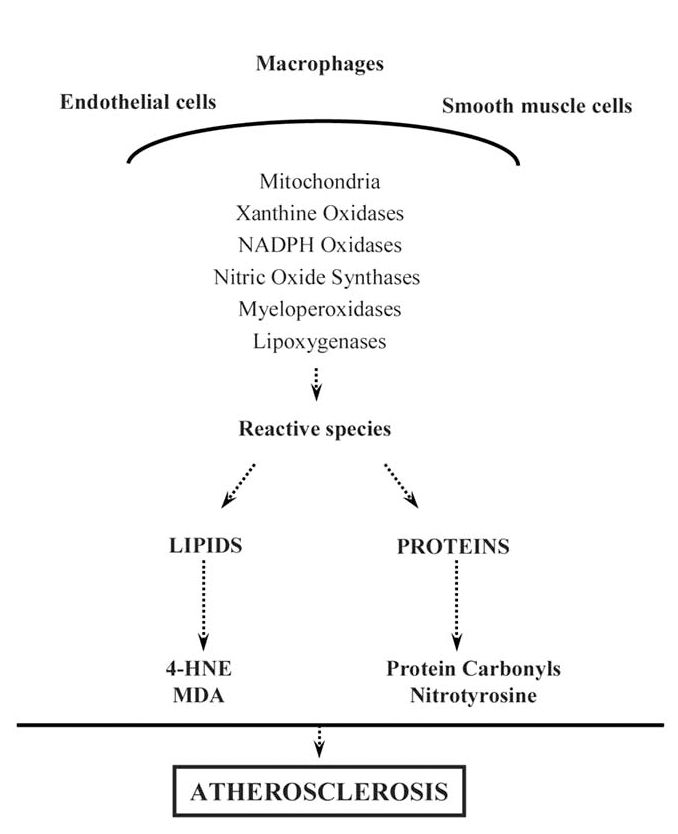
**Main pathways during the development of atherosclerosis.** Macrophages, endothelial cells and smooth muscle cells are the cells cause reactive species formation during the atherosclerotic process. Reactive species damage cellular constituents and several products are formed those can be used as biomarker in the atherosclerosis.

Redox signaling process is shown to be involved in the pathogenesis of atherosclerosis besides several different hypotheses [18]. Lipid peroxidation and LDL oxidation induced by RS are the early events in atherosclerotic lesion formation [19-22]. There is now a consensus that atherosclerosis represents protein oxidation process in the vascular wall besides lipid oxidation [1]. Mostly macrophages are thought to be the source of ROS formation in the vessel wall but also other cells like endothelial, smooth muscle and adventitial cells produce ROS in the vessel wall (Fig. **1**) [19, 23] .

Ox-LDL modulates atherosclerosis biology by cell damage induction, proliferation of smooth muscle cells, foam cell formation, chemotaxis of leukocytes and secretion of inflammatory mediators. Since oxidation of LDL is the main oxidative modification, high plasma levels of native LDL is a risk factor for the progression [24]. Ox-LDL uptake by macrophages is easier compared to non-oxidized LDL. It is known that hypercholesterolemia stimulates ROS formation from smooth muscle cells and this also leads to increased oxidation of LDL [21]. Ox-LDL includes oxidative agents such as aldehyde end products of lipid peroxidation of polyunsaturated fatty acids like HNE, derived from phospholipids, mono-, di-, and triacylglycerols, or cholesteryl esters (CEs), as well as cholesterol oxidation products. These oxidized lipids *via* their fibrogenic, apoptotic, coagulant, and inflammatory effects, contribute to progression of atherogenic lesions. Oxidation of LDL changes the composition of the particle. *In vitro* oxidation of LDL can be achieved by copper incubation [25] and incubation with culture medium from 15-lipoxygenase-overexpressing fibroblasts [26]. Oxidation of the lipid moiety of lipoproteins and formation of lipoperoxides transferred to LDL cause oxysterol formation. The unsaturated fatty acyl chains of phospholipids, CEs, and triglycerides are oxidized most readily, and a significant proportion of the unsaturated acyl chains are also oxidized to hydroperoxides, isoprostanes, and more AGEs [27]. Cholesterol and saturated fatty acids react more slowly, and a small proportion of cholesterol is converted to oxysterols, initially 7-hydroperoxycholesterol. Oxysterols, 27-carbon products of cholesterol oxidation, are possible reactive mediators of structural and functional changes of the vascular wall, which are affected by the atherosclerotic process [28]. ApoB, the dominant apoprotein of LDL, which is highly glycosylated, is subject to both direct oxidative modification and reaction with products of lipid oxidation.

Main reactive oxygen species implicated in cardiovascular diseases are superoxide (O_2_˙¯), hydroxyl (OH˙) and hydrogen peroxide (H_2_O_2_) and reactive nitrogen species are NO and peroxynitrite. While superoxide and hydroxyl radicals are more reactive, hydrogen peroxide is more membrane permeable. As the basic mechanism, these oxygen species are converted to each others by several mechanisms. O_2_˙¯ is dismutated nonenzymatically or enzymatically by superoxide dismutase (SOD) to H_2_O_2_. Also various enzymes located in the plasma membrane, cytosol, peroxisomes and mitochondria catalyse ROS formation.

### Sources of Reactive Oxygen Species

Enzymatic sources of O_2_˙¯ include NADPH oxidases, xanthine oxidase, cyclooxygenase, lipoxygenase, cytochrome P-450 enzymes, uncoupled NOSs, phagocytic myeloperoxidase system and mitochondrial electron transport chain [29-31].

Mitochondria is an important source of O_2_˙¯. In the electron transport chain, mainly respiratory electron carriers induces O_2_˙¯ formation. Complex I (containing the flavin mononucleotide [FMN] and iron sulfide [FeS] potential O_2_˙¯-producing sites) was, for a long time, considered one of two major sites of O_2_˙¯ production. The second major site of mitochondrial O_2_˙¯ production is believed to be ubiquinone-Complex III [32]. Ox-LDL was found to induce O_2_˙¯ formation in mitochondria [33]. In endothelial cells, 4-HNE was shown to inactivate proteins containing reactive thiols, such as 2-oxoglutarate dehydrogenase and pyruvate dehydrogenase [34], and thereby inhibit complex I-dependent (NADH-linked) respiration [35].

### Xanthine Oxidase (XO)

XO is located in the endothelium of various organs and causes O_2_˙¯ production during the catalysis of the oxidation of hypoxanthine to urate. Tissue distribution of XO is an important factor of O_2_˙¯ induced injury. XO and xanthine dehydrogenase are two forms of the enzyme and in basal conditions dehydrogenase form is present in the tissues. Either through oxidation or by proteolytic cleavage of xanthine dehydrogenase, XO is formed. The levels of plasma-circulating XO and the ability of circulating XO to bind vascular cells of various organs increase during some pathological states such as reperfusion injury, hepatitis, adult respiratory distress syndrome and atherosclerosis [30]. In experimental animals with hypercholesterolemia it is capable of producing increased amounts of active radicals leading directly to reduced nitric oxide (NO) activity [36]. Additional facts that support the role of xanthine oxidase in the process of atherogenesis are the following: (i) in patients with coronary syndrome the levels of this enzyme were found to be increased-the same applies to NAD(P)H; and (ii) in young asymptomatic patients with familial hypercholesterolemia the increased activity of the enzyme is an early event [37].

### NADPH Oxidase

NADPH oxidase, is a multiple subunit electron transport system, was discovered in neutrophils where it catalyzes one electron reduction of oxygen to produce O_2_˙¯ using NADPH as the electron donor during phagocytosis and plays role in immune protection with its bactericidal activity [31, 38, 39]. This enzyme system plays key role in generating ROS in fibroblasts, vascular smooth muscle cells and endothelial cells besides phagocytic cells. The NADPH oxidase subunits are shown to be present in human blood vessels including atherosclerotic coronary arteries, in veins and mammary arteries with coronary artery disease, which strengthens the importance of the molecular regulation of the enzyme in cardiovascular diseases [6, 40]. NADPH oxidase activity in non-phagocytic cells, such as cardiovascular cells, is acutely increased by diverse pathophysiological stimuli including: (i) G- protein-coupled receptor agonists, e.g. angiotensin II and endothelin-1; (ii) cytokines, e.g. (tumour necrosis factor-α) TNF-α and (transforming growth factor-β) TGF-β; (iii) growth factors, e.g. thrombin, (vascular endothelial growth factor) VEGF and insulin; (iv) ‘metabolic’ factors, e.g. ox-LDL, nonesterified (free) fatty acids and glycated proteins; (v) hypoxia-reoxygenation or ischaemia-reperfusion; and (vi) mechanical stimuli, e.g. oscillatory shear [3].

The phagocytic NADPH oxidase consists of a membrane-associated cytochrome b558 that comprises a large subunit, gp91^*phox*^ (‘phox’ being derived from phagocytic oxidase), and a small one, p22^*phox*^. Besides these, there are at least three cytosolic subunits (p47^*phox*^, p67^*phox*^ and p40^*phox*^) and a low-molecular-weight G protein (Rac1 or Rac2). p47^*phox*^, p67^*phox*^ and gp91^*phox*^ (NOX_2_) present on phagocytic NADPH oxidase have been identified in vascular cells. However, several studies have confirmed that p22^*phox*^ is present in all NADPH oxidase systems and that this subunit is essential for the functionality of the enzyme. Upon cell stimulation, p47^*phox*^ becomes phosphorylated on multiple sites with several kinases (protein kinase C, protein kinase A, or mitogen activated protein kinase) and the cytosolic subunits form a complex which migrates to the membrane where it binds to the cytochrome b558. Then electrons are transferred from the substrate, NADPH, to O2, leading to O_2_˙¯ generation [19, 41]. Phosphorylation and translocation of p47^*phox*^ allows its interaction with p22^*phox*^ and facilitates the binding of p67^*phox*^ to cytochrome b558 [31]. Also another key posttranslational modification involved in oxidase activation is Rac activation.

Several homologues of gp91^*phox*^, have recently been reported to be expressed in nonphagocytic cells. Other members of the NOX family comprise of NOX1,NOX2 NOX3, NOX4 and NOX5, as well as larger and more complex homologues termed DUOX1 and DUOX2 [38]. NOX1 to 5 are 65-kDa core proteins, whereas DUOX 1 and 2 are 175- to 180-kDa proteins that have a domain homologous to gp91^*phox*^ as well as an additional peroxidase domain. Using this new terminology, NOX2 represents the neutrophil gp91phox. The first homologue of gp91^*phox*^, namely NOX1, was found to have significant proliferative activity and was also therefore known by the alternative term mitogenic oxidase or MOX-1 [30].

Functionally endothelial NADPH oxidase shares some but not all of the characteristics of neutrophil NADPH oxidase. One major difference is that endothelial NADPH oxidase continuously generates a low level of O_2_˙¯ even in unstimulated cells, although its activity can be further increased by several agonists. However, neutrophil NADPH oxidase primarily produces O_2_˙¯ when the cells are stimulated. In regard to the isoform of NOXs, gp91^*phox*^ (NOX2) has been considered as the major isoform of NOX proteins in vascular endothelial cells [42, 43]. The functional role of this NOX isoform has been confirmed by decrease in phorbol ester-induced O_2_˙¯ production and endothelium-dependent relaxation in gp91^*phox*^¯/¯ mice [43]. In addition to gp91^*phox*^, NOX4 mRNA is also detectable in endothelial cells. It appears that NOX4-dependent oxidase functionally contributes to the basal O_2_˙¯ production in endothelial cells [44].

### Nitric Oxide Synthase (NOS)

Ox-LDL also activates nitric oxide synthase and increases production of NO as a key regulator of vasodilatation. Multiple interactions of NO˙ with oxidizing lipids could lead to either vascular protection or potentiation of inflammatory vascular injury. Ox-LDL increases O_2_˙¯ production in endothelial cells and decreases the bioavailability of NO through a process involving lectin-like ox-LDL receptor (LOX-1). Low levels of NO˙ generated by endothelial NOS (eNOS) can terminate lipid radicals and inhibit lipoxygenases, which would be protective. But elevated levels of NO˙, for example, after inducible nitric oxide synthase (iNOS) expression in inflammation, can be converted to prooxidant species like peroxynitrite (ONOO˙) and NO2 [45]. In the presence of available O2 radicals, ox-LDL may contribute to ONOO˙ formation, which can potentiate inflammatory injury of vascular cells. A role for ONOO^#^ in initiating lipid oxidation in atherosclerosis has been suggested [46]. The reduction of endothelial-produced NO and O_2_˙¯ is able to blunt normal endothelial dysfunction as a result of the decreased endothelial NO production. The increased production of ROS reduces the production and consequently the bioavailability of NO, leading to vasoconstriction, platelet aggregation and adhesion of neutrophils to the endothelium [47].

### Myeloperoxidases (MPO)

This enzyme is uses H_2_O_2_ for the production of more powerful oxidative substances by activated phagocytes. This enzyme, through NADPH, leads to the production of hypochlorous acid (HOCl) and its analogs (substances related to endothelial injuries due to the action of H_2_O_2_) [48]. It is considered to participate in the process of atheromatosis by the induction of oxidative modifications in low and high density lipoproteins [49]. This hypothesis is consistent with the results of clinical trials, according to which the levels of MPO and its products are elevated in patients with coronary syndrome. In contrast to human lesions, these oxidative products are absent in experimental animals with apolipoprotein E and LDL-receptor deficiency [21]. The three mechanisms through which myeloperoxidase participates in oxidative modifications are NO consumption, LDL oxidation, and reaction with L-arginine for the production of NO synthase inhibitors. All of these are dependent on H_2_O_2_. Also immunohistochemical studies have proved the presence of myeloperoxidase and HOCl in atherosclerotic lesions [50].

High density lipoprotein (HDL) isolated from patients with cardiovascular disease contains elevated levels of 3-chlorotyrosine and 3-nitrotyrosine, which are two characteristic products of MPO, enzyme secreted by macrophages [51]. MPO-dependent oxidation of specific amino acids, mainly tyrosine and methionine residues of apoA-I, impairs its ability to remove excess cellular cholesterol *via* the ATP-binding cassette transporter A1 (ABCA1) pathway [52]. MPO also generates HOCl like H_2_O_2_ and other ROS, which is also secreted by macrophages [53]. Enzymatically active MPO was found in human atherosclerotic lesions [49], and lipoproteins that have been modified by HOCl have been detected in advanced human atherosclerotic plaques. Tyrosylated HDL is more potent than native HDL in removing cholesterol from lipid-laden fibroblasts and macrophages *in vitro*. This process does not appear to involve passive cholesterol desorption from the cell-surface membranes [54], which suggests the possibility that tyrosylated HDL promotes reverse cholesterol transport by interacting with ABCA1 in macrophages and perhaps other peripheral tissues more efficiently than native HDL [55, 56]. Enzymatically active MPO and elevated levels of dityrosine, marker for protein oxidation by tyrosyl radicals, have been detected in human atherosclerotic plaques [49].

### Lipoxygenases

Lipoxygenases are a family of iron-containing enzymes that catalyse the dioxygenation of polyunsaturated fatty acids in lipids containing a cis,cis-1,4- pentadiene structure, creating a family of biologically active lipids, such as prostaglandins, thromboxanes and leukotrienes, which participate in inflammatory reactions and increase the permeability of vessels. In experimental models, 15-lipoxygenase was shown to induce LDL oxidation by enzymatic and nonenzymatic reactions. Experimental animals with an absence of the 15-lipoxygenase gene or reduced expression of 5-lipoxygenase are protected from lesions like those found in animals with apolipoprotein E and LDL-receptor deficiency [57]. Clinical data demonstrate that various genotypes of 5-lipoxygenase promoter are found in patients with atherosclerotic lesions or inflammation [21].

#### Effects of Reactive Species on Signaling Mechanisms

O_2_˙¯ anion, in addition to mediate LDL oxidation, may contribute to the pathogenesis of atherosclerosis in various ways [7]. O_2_˙¯ inactivates endogenous vasodilatator, endothelium derived NO, thereby promotes vasoconstriction. Impairment of NO function by O_2_˙¯ also results in vascular smooth muscle cell proliferation and migration [58]. It was shown that incubation of vascular smooth muscle cells with H_2_O_2_, induced the expression of VEGF, confirming the role of ROS in neovascularization in atherosclerotic plaques and restenotic lesions [59]. Redox molecules including ROS and RNS possess redox potential, play important role in the maintenance of cardiac homeostasis by acting through specific signal transduction pathways [60, 61]. Key components in atherogenesis including signaling molecules such as redox sensitive transcription factor NFκB activation and adhesion molecules such as selectins, vascular cell adhesion molecule-1 (VCAM-1) and intercellular adhesion molecule-1 (ICAM-1) and chemokines such as monocyte chemoattractant protein-1 (MCP-1) expressions in the vascular endothelium are known to be increased by RS [58]. Expression of adhesion molecules and MCP-1 are also key steps for the monocyte adhesion and emigration to form macrophages and foam cells [30].

Macrophage colony-stimulating factor (M-CSF) is an important factor regulating the survival, proliferation, differentiation, and chemotaxis of macrophages. During early myeloid differentiation, M-CSF synergizes with other growth factors and interleukin-3 to produce mononuclear phagocyte progenitor cells. After this initial differentiation process, M-CSF by itself can regulate the proliferation and differentiation of mononuclear phagocyte progenitor cells to monocytes, macrophages, and osteoclasts and also supports survival and activity of fully differentiated macrophages. The receptor for M-CSF (M-CSF-1R) is expressed in mononuclear phagocytes and antigen presenting cells (APCs), which can be regarded as a specialized adaptive state rather than a separate lineage. M-CSF was considered as an alternative marker of macrophages, whereas APCs differentiate through the action of granulocyte-macrophage-CSF (GM-CSF), IL-4, and prostaglandin E2 [62]. M-CSF cooperates with the receptor activator of NFκB ligand (RANKL) to regulate the differentiation of mononuclear phagocytes toward osteoclasts [63]. M-CSF also enhances cytotoxicity, ROS (e.g., superoxide radical, peroxynitryl-, hydroxyl-radical and hydrogen-peroxide production), as well as phagocytosis, chemotaxis, and cytokine production in monocytes and macrophages [64]. M-CSF-mediated signaling involves many cytoplasmic molecules like c-Src, which is linked with c-Cbl and targets the Vav family members of guanine nucleotide exchange factors (GEFs), which in turn activate Rac-1 as a constituent of activated NADPH-oxidase. Alternatively coregulatory signaling pathways like integrin signaling (e.g., ανβ3, αMβ2) also target Vav. Ox-LDL is directly mitogenic for macrophages and smooth muscle cells, and stimulates the release of MCP-1 and M-CSF from endothelial cells and the production of many inflammatory mediators (e.g., endothelin-1) from other vascular cells and chemotactic for monocytes/APCs and T cells [65].

The effects of ox-LDL on NFκB may be biphasic as concentration dependent. Normally, it activates NFκB and upregulates the expressions of adhesion molecules, tissue factor and LOX-1. In high concentration, ox-LDL inhibits NFκB activation triggered by inflammatory agents such as lipoxygenases and therefore exert immunosupressive effect [66]. HNEs were shown to activate MAPK in endothelial cells either by directly interacting with PKC or through activation of the EGF receptor [67]. Transcription factor AP-1, plays role in the regulation of TGF-β1, procollagen type I, platelet-derived growth factor-AA (PDGF-AA), MCP-1, and cyclooxygenase-2 (COX-2) expressions, were activated by HNE [68].

The presence of foam cells in the atherosclerosis process confirms the importance of CD36 scavenger receptors. CD36 has an important role in the intake of ox-LDL by macrophages in the arteria walls and long chain fatty acids into the cells. Following binding of ox-LDL to CD36 receptor, lyn kinase, a src protein tyrosine kinase, is activated. This activation induces mitogen ERK kinase kinase 2 (MEKK2) and c-jun N-terminal kinase (JNK) activation and phagocytosis of proatherogenic ox-LDL [69]. Studies carried out in murine models showed that inhibition of JNK causes a decrease in ox-LDL uptake [70, 71]. CD36, was shown to be upregulated by PKC and PPARγ pathway which are common signaling mechanisms for IL4 and ox-LDL [72]. CD36 scavenger receptor expression was shown to be increased in ox-LDL treated aortic smooth muscle cells in culture [73]. It has been shown as *in vivo* that hypercholesterolemia increases foam cell formation and atherosclerosis by increasing CD36 mRNA expression and PKC activity in rabbits [74-76].

Fatty acids and their oxidation products activate the nuclear orphan receptors PPARs. These are ligand-activated transcription factors that play an important role in obesity-related metabolic diseases such as high triglyceride/low-HDL syndromes, insulin resistance, and coronary artery disease [77]. PPARs bind, on heterodimerization with RXR, to specific peroxisome proliferator response elements (PPREs) in the promoter of target genes, thus regulating the transcription of these genes. PPARs consist of isoforms α, γ, and δ with distinct expression patterns and biologic activities. PPARs are expressed in atherosclerotic lesions and have been shown to affect transcription of genes in vascular endothelial cells, smooth muscle cells, monocytes, and monocyte-derived macrophages. PPAR-α induces an increase in ROS, which leads to induction of NADPH-oxidase activity in macrophages and results in the generation of LDL species with PPAR-α activation properties [78]. PPAR-γ expression is significantly increased on monocyte-to-macrophage differentiation, and PPAR-γ protein is present at high levels in monocytes and macrophage-derived foam cells of atherosclerotic lesions [79, 80] and in circulating human monocytes, where its activation increases the expression of macrophage-specific markers, such as CD14 and CD11b, which are constituents of lipid rafts. Treatment of macrophages with ox-LDL *in vitro* induces mRNA expression of PPAR-γ and LXR-α, a direct transcriptional target of PPAR-γ. Internalization of ox-LDL provides the cell with activators of PPAR-γ, such as the oxidized fatty acids 9-and 13- hydroxyoctadecadienoic acid (9- and 13-HODE), as well as with activators of LXRs such as 27- and 25-hydroxycholesterol [81, 82]. PPAR-γ ligands can also be produced locally in atherosclerotic lesions through the oxidation of fatty acids by 12/15 lipoxygenase [83]. Arachidonic acid metabolites derived from the cyclooxygenase and lipoxygenase pathways [e.g., 15-deoxy-Δ-12, 14-prostaglandin J2 (PGJ2), and 15-hydroxyeicosatetraenoic acid (15-HETE) [82, 84] activate also PPAR-γ. PPAR-γ activators inhibit the expression of MMP-9 in human macrophages [85] and vascular smooth muscle cells [86] and the production of the inflammatory cytokines TNF-α, IL-6, and IL-1β by activated monocytes [87]. The induction of the scavenger receptor CLA-1/SR-BI is inhibited by PPAR-γ in human macrophages [88]. Activation of PPAR-γ has been shown to enhance CD36 expression of macrophages, which may indicate that PPAR-γ could stimulate uptake of ox-LDL and contribute to foam cell formation [82]. These CD36 effects may be compensated through the activation of LXRα,which promotes cholesterol efflux by modulating expression of ABCA1 and apoE [89]. Silverstein and Febbraio [90] showed a decrease in CD36 mRNA expression as a result of inhibition in the transcriptional activity of PPARγ following phosphorylation by TGF-β. Tontonoz et al. [80] confirmed the relation of PKC with PPARγ induction and CD36 expression with the results showing that diacylglycerol and ingenol as PKC activators regulate the mRNA expression of CD36. Additionally rosiglitazone as a PPARγ agonist was shown to increase CD36 expression in macrophages [91]. Leonarduzzi et al. [92] tested the effects of non-oxidized and oxidized cholesterol on monocytic cell differentiation and foam cell formation and found out that while oxysterols stimulated CD36 expression and synthesis in human U937 promonocytic cells, nonoxidized cholesterol did not exert any effect. When investigated in detail, the upregulation of CD36 was found to be based on the activation of protein kinase Cδ, extracellular signal-regulated kinase 1/2 (ERK1/2) and PPARγ.

When cholesterol acceptors such as HDLs are present, cholesterol efflux from macrophages is accelerated, which prevents foam cell formation. The ATP-binding cassette transporters (ABCs) ABCA1 and ABCG1 facilitate transport of free cholesterol and cholesterol/phospholipid complexes (UC/PL) across cell membranes in cholesterol efflux pathways. During this process, ABCA1 promotes nascent discoidal pre-β-HDL particle formation from lipid poor apoA-I. In humans and mice, apoA-I is produced primarily in the liver and intestine. Extracellular sources of apoA-I have been shown to increase cholesterol efflux from macrophages *in vitro* [93] and are considered to be necessary for the activation of cholesterol efflux through the ABCA1 pathway [94]. The Rho family GTPase Cdc42 directly interacts with ABCA1 to control filopodia formation, actin organization, and intracellular lipid transport [95]. Vesicular transport processes involving different interactive proteins like β2-syntrophin are involved in cellular lipid homeostasis controlled by ABCA1 [96].

Cell-adhesion molecules such as ICAM-1, present within the endothelium and increases monocyte adhesion, is upregulated by lysophosphatidylcholine formation following phophatidylcholine degradation. This formation can induce mitochondrial ROS formation through Ca^2+^-dependent process and leads to the activation of ERK/MAPK pathway. The mechanism is explained as the interaction between the Ca^2+^-dependent mitochondrial dehydrogenases and complex I [97].

Heme/iron oxidative damage can be promoted by increased heme/iron levels released into the plasma from damaged red blood cells that are removed by binding to hemopexin and haptoglobin and subsequent cellular uptake *via* CD163 cysteine-rich SRs into monocytes and macrophages. Heme is oxidized and rapidly converted into hemin. One portion is removed by hemopexin, but the rest interacts with cell membranes [98] and with circulating LDL and HDL [99]. Accumulation of hemin, however, triggers an oxidative-stress response that promotes heme degradation by HO-1 into bilirubin, iron, and CO. Because overexpression of HO-1 has been found to protect animals from atherosclerotic lesions, it is suggested that hemin is a risk factor for atherogenesis and that HO-1 can protect cells from oxidative damage [99]. Hemoglobin promotes formation of ROS and catalyzes LDL oxidation and covalent cross-linking of the LDL protein apoB through the globin radical [100].

Tyrosine kinases are known to affect vessels in several ways. In a study, H_2_O_2_ was shown to increase the phosphorylation of tyrosine kinases and lead to stronger binding of neutrophil cells on endothelium and alteration of vessel permeability [47].

In advanced lesions, macrophages become apoptotic. Apoptosis is induced with the accumulation of free cholesterol mainly in the endoplasmic reticulum membrane and alters the function of integral endoplasmic reticulum membrane proteins. These chain of events induces the endoplasmic reticulum stress signal transduction pathway also known as the unfolded protein response. Evidence from *in vivo* studies suggests that this pathway plays important role in atherosclerotic lesions [101].

## ANTIOXIDANT SYSTEMS IN ATHEROSCLEROSIS

A redox couple is a molecule or enzyme that switches between reduced and oxidized forms. Two of the most important redox couples are thioredoxin (Trx) and glutathione (GSH). Trx is a small (12 kDa) multifunctional protein carrying two cysteines that reversibly switches from dithiol to disulfide [Trx(SH)2 to TrxS2]. TrxS2 is generally reduced by NADPH and flavoprotein thioredoxin reductase (TrxR). Trx ubiquitously expressed in all organs including heart and is deeply involved in the protection of cardiomyocytes by its antioxidant, antiapoptotic as well as anti-inflammatory properties. Trx is localized in both the cytosol and the nucleus [18]. Plasma or serum Trx levels can be easily determined by utilizing the ELISA assay [102]. Moreover, in the various cardiovascular disease conditions, the expression level of Trx is altered either in organ or in plasma or in both. The uptake of ox-LDL by macrophages highly induces Trx expression [103]. Highly elevated plasma or serum Trx levels are reported in patients with diabetes mellitus, especially with diabetes mellitus type 2 or with glucose intolerance, and patients with hypertension, hypercholesterolemia, and atherosclerosis, all of which are major risk factors for cardiovascular diseases [103, 104]. By providing electrons, Trx and GSH maintain intracellular proteins in a reduced state. As part of the cellular defence against oxidative stress, expression of different genes of the GSH and Trx systems is increased when cells are exposed to ROS [14]. ROS, RNS, and electrophilic lipids contribute to the posttranslational modification of protein thiols (protein-SH) to form S-nitrosothiols (SNOs). This is a prevalent posttranslational protein modification involved in redox-based cellular signaling. Under physiologic conditions, protein S-nitrosylation and SNOs provide protection preventing further cellular oxidative and nitrosative stress. Conversely, increased oxidative stress and the resultant dysregulation of NO are implicated in the pathogenesis of cardiovascular diseases [105]. Many intracellular redox-sensitive processes, including synthesis of DNA precursors by ribonucleotide reductase, transcription factor regulation, and cellular growth [106], are modulated by the Trx system, composed of Trx, TrxR, and NADPH. Because the many antioxidant and regulatory roles of cytosolic Trx are dependent on the activity of cytosolic TrxR, this selenoenzyme together with Trx is increasingly being recognized as an essential component for cellular redox control and antioxidant defense [14, 106]. The ubiquitous 55-kDa selenoprotein TrxR1 was found upregulated in human atherosclerotic plaques and expressed in foam cells [107]. TrxR1 mRNA in human monocyte-derived macrophages dose-dependently increases with ox-LDLs, but not with native LDLs.

Specific protein disulfide targets for reduction by Trx are protein disulfidisomerases (PDI) and Trx is also a specific electron donor for many peroxiredoxins, which are important for the reduction of peroxides and have generated recent interest for their potential to regulate signaling pathways. In macrophage-derived foam cells on ox-LDL stimulation, peroxiredoxin I (Prx I) plays a dual role. As an antioxidant, induction of Prx I during treatment with ox-LDL led to improved cell survival with a decrease in ROS. Additionally, activation of p38/MAPK was dependent on the upregulation of Prx I. Therefore, Prx I in macrophage-derived foam cells could be considered both an antioxidant and a regulator of oxidant-sensitive signal transduction [108].

GSH is a cysteine-containing tripeptide that reversibly forms a homodimer, GSH disulfide (GSSG). The GSH/glutaredoxin (GR) system plays a critical role in protecting macrophages from ox-LDL-induced cell death, and the disturbance of this system renders macrophages susceptible to oxidative stress-induced cell injury [109].

GSH and Trx are involved in cardiovascular disorders, and low serum concentration of GSH is associated with coronary artery disease [110], whereas elevated serum Trx levels are correlated with acute coronary syndrome [111]. Ox-LDLs are able to modulate the intracellular GSH level through the induction of enzymes in GSH synthesis [112], and macrophages treated with ox-LDL have increased cellular content of GSH [113] and activate the antioxidative Trx and GSH systems and TrxR1, and GSH reductase [114]. Pharmacologic depletion of cellular GSH enhanced ox-LDL cytotoxicity to human monocytes and macrophages [115], suggesting that GSH may protect macrophages from ox-LDL cell injury. Glutaredoxin (thioltransferase), which acts as an electron carrier in the glutathione-dependent synthesis of deoxyribonucleotides by the enzyme ribonucleotide reductase, was found highly expressed in macrophages invading atherosclerotic plaques [103].

## LIPID RAFT REDOX SIGNALING IN ATHEROSCLEROSIS

As a mosaic of different compartments or domains, the biologic membranes can form a number of types of subdomains due to the interaction between membrane components. Lipid rafts (LRs) are originally defined as sphingolipid- and cholesterol-rich microdomains in the plasma membrane that play a role in a number of signaling processes involving specific receptors [116]. A growing body of evidence supports the notion that lipid rafts play a crucial role in the redox signaling that regulates the pathophysiology of many degenerative diseases [117]. It is also known that distinct cholesterol- and sphingolipid-rich membrane rafts is importantly involved in transmembrane signaling in a variety of mammalian cells [118, 119]. The formation of LR signaling platforms with aggregation of different signaling molecules may represent one of important mechanisms determining the variety of transmembrane signaling; it also robustly amplifies signals from activated receptors. Among these LR signaling platforms, it is also reported that some large redox signaling molecules are also aggregated or recruited into LR clusters and subsequently produce superoxide and other ROS [120-122]. This type of LR signaling platforms with ROS production has been referred to as LR redox signaling platforms [121]. This LR signaling mechanism has also been reported to play important roles in the normal regulation of many other cell or organ functions and in the development of different pathological conditions of different cells or organs.

LRs are considered to be an important signaling component in the cell membrane [116, 119]. LRs consist of dynamic assemblies of cholesterol and lipids with saturated acyl chains that include sphingolipids and glycosphingolipids in the exoplasmic leaflet of the membrane bilayer. In addition, phospholipids with saturated fatty acids and cholesterol in the inner leaflet are important elements for LRs. By interdigitation and transmembrane proteins, the long fatty acids of sphingolipids in the outer leaflets couple the exoplasmic and cytoplasmic leaflets, which form a very stable and detergent-resistant membrane structure [119, 123]. This stable structure is one of the most basic features of LRs. Different from this stable membrane structure, a large portion of cell membrane lipid (60-80%) is more fluid, as it mostly consists of phospholipids with unsaturated and kinked fatty acid chains, as well as cholesterol. Another interesting feature of these membrane LRs is their capacity of including or excluding proteins to variable extents when cells respond to different physiological or pathological stimuli. Many proteins have been demonstrated to have LR affinity such as glycosylphosphatidylinositol (GPI) anchored proteins, doubly acylated proteins, cholesterol-linked proteins, and some other transmembrane proteins, including ion channels, tyrosine kinases, and different membrane exchangers or transporters [119, 124].

Actually two types of lipid rafts are identified in the biologic membranes: caveolae and noncaveolae lipid rafts. Two major models are commonly cited or accepted currently to describe the nature or behavior of lipid rafts. In the first model, lipid rafts are considered relatively small structures enriched in cholesterol and sphingolipids within which associated proteins are likely to be concentrated [116]. In this sphingolipid-enriched lipid raft, the most prevalent component of the sphingolipid fraction in the cell membrane is sphingomyelin (SM), which is composed of a highly hydrophobic ceramide moiety and a hydrophilic phosphorylcholine headgroup. The tight interaction between the cholesterol sterol ring system and the ceramide moiety of SM promotes the lateral association of sphingolipids and cholesterol and thereby the formation of distinct microdomains. In these microdomains, cholesterol exerts a stabilizing role by filling the voids between the large and bulky glycerosphingolipids. It is this cholesterol-SM interaction that determines a transition of these microdomains into a liquid-ordered or even gel-like phase, which is a unique characteristic of lipid rafts. Other domains of the cell membranes primarily exist in a more fluid liquid-disordered phase because of the absence of this cholesterol-SM interaction [125].

Caveolae, 50-100 nm invaginations of the plasma membrane, are a subset of lipid rafts enriched in sphingolipids and cholesterol. The caveolae can selectively sequestrate membrane-targeted proteins and create a unique signaling microdomain, thereby controlling transmembrane signaling. Caveolae are characterized by the presence of caveolins, which distinguishes caveolae from other lipid raft domains. At least three caveolin isoforms of molecular weights between 22 and 24 kDa have been identified: caveolin 1 and caveolin 2 are abundant in most cell types, while caveolin 3 is specific to muscle cells [126]. Caveolin 1, a substrate for nonreceptor tyrosine kinases including Fyn, Abl, and Src, acts as a scaffolding protein and can be phosphorylated on tyrosine 14 by these kinases in response to external stimuli such as ROS. Such tyrosine phosphorylation activates the downstream signaling targets and thus serves as a crucial step for intracellular signaling occurring within caveolae. Sphingomyelinase (SMase)-dependent cleavage of sphingomyelin resulting in the formation of ceramide appears to play a crucial role in lipid raft formation [127]. Sphingomyelin hydrolysis is catalyzed by SMases that hydrolyze the phosphodiester bond of sphingomyelin, yielding ceramide and phosphocholine. There is convincing evidence that ceramide performs its signaling function from within the lipid rafts, ordered sphingolipid- and cholesterol-rich lipid domains [128], which can function as an ordered support for receptor-mediated signaling events.

Initiation of intracellular signaling cascades is associated with aggregation or reduction of cell surface receptors through LR clustering in the plasma membrane [120, 129]. These receptors aggregated in LR clusters are many, including T-cell receptor/CD3 complex, B cell receptors, CD2, CD40, CD44, L-selectin, insulin receptors, or integrins, which transfer the signal to these transmembrane signaling proteins or proteins in inner leaflets of the cell membrane when they aggregate within LR clusters. This completes the transmembrane signaling process [130, 131]. Recent studies have indicated that several death receptors, including tumor necrosis factor receptor (TNFR), Fas, and DR 4 and 5 produce their detrimental effects through this mechanism [119, 132]. During LR clustering, aggregated receptors or other signaling molecules could be either constitutively located in LRs or translocated by trafficking or recruitments upon stimulation [133, 134]. This dynamic clustering of lipid microdomains may represent a critical common mechanism in transmembrane signal transduction. It has been reported that clustered LRs contain different compositions of proteins, constituting platforms or macrodomains that form a new mixture of molecules, including different signaling molecules and crosslinkers or enzymes [116, 119]. The formation of LR platforms activates, facilitates, or amplifies signal transduction. There is considerable evidence that LR clustering could be formed as a ceramide-enriched membrane platform and that ceramide production or enrichment is through sphingomyelinase (SMase)-catalyzed cleavage of choline from sphingomyelin (SM) in individual LRs [135, 136]. However, ceramide-enriched membrane platforms might also be formed without existence of classically-defined LRs simply through a fusion of several ceramide molecules. These ceramide molecules could come from LRs or other membrane fractions.

These lipid-rich microdomains (lipid rafts) of the cell membrane are central to the understanding of cellular lipid homeostasis and the consequences of lipid loading on cell function. They are ceramide, which is induced by ox-LDL through enhancement of neutral and acidic sphingomyelinase (SMase) activity [137], leads to coalescence of submicroscopic rafts into large ceramide membrane macrodomains [135]. These macrodomains may serve as platforms for protein concentration and oligomerization, transmitting signals across the plasma membrane. ceramide then activates a variety of diverse protein kinase- and protein phosphatase-dependent signaling pathways, which in most cases suppress cell growth or promote programmed cell death or both [135]. In addition, ceramide is a ligand for apoE, which binds more avidly to ceramide-rich microdomains on sphingomyelinase-treated liposomes [138]. Together with the stimulation of heparan sulfate proteoglycan (HSPG) and low-density lipoprotein receptor elated protein (LRP)-mediated uptake by macrophages through ceramide and apoE, which is crucial for foam cell formation [139], a function of ceramide-rich microdomains in apoE-dependent metabolism is suggested.

Lipid rafts can be disrupted by cholesterol depletion, whereas cholesterol enrichment stabilizes the formation of lipid rafts [116]. E-LDL preferentially increases cellular cholesterol, whereas ox-LDL increases cellular ceramide content because of a higher mRNA expression of acid and neutral sphingomyelinase (SMase), neutral SMase activation-associated factor, and glucosylceramidase during ox-LDL loading [3]. E-LDL and ox-LDL differentially influence membrane-microdomain formation in human macrophages and thereby differ in their regulation of macrophage effector functions during atherogenesis. Glycosphingolipids, as constituents of lipid rafts and especially ceramides, are ligands for apoE, and apoE binds more avidly to ceramide-rich microdomains on sphingomyelinase-treated liposomes [138]. This indicates a function of ceramide-rich microdomains in apoE-dependent metabolism. The generation of ceramide in the plasma membrane by SMases may stimulate HSPG and low-density lipoprotein receptor-related protein (LRP)-mediated uptake by macrophages, which is catalyzed by apoE, and plays a crucial role in tissue remodeling and foam cell formation [139]. Concerning the relation of atherogenic LDL and lipid rafts in human aortic endothelial cells, ox-LDL causes the disappearance of the lipid-raft marker GM1 from the plasma membrane. Exposure to ox-LDL may result in the disruption or redistribution of lipid rafts, which in turn induces stiffening of the endothelium, an increase in endothelial force generation, and the potential for network formation [140].

A significant amount of eNOS, which generates NO in the endothelium, is found in caveolae. Caveolae are flask-shaped invaginations of the plasma membrane that are coated with the protein caveolin, which can function as a negative regulator of eNOS [141]. They contain proportionately small amounts of phospholipids and large amounts of cholesterol, sphingomyelin, and glycosphingolipids as well as SR-BI and CD36. Ox-LDL causes an efflux of caveolae cholesterol out of the cell and onto ox-LDL, leading to a redistribution of eNOS and caveolin to an intracellular membrane [142]. This requires the presence of ox-LDL binding to CD36, whereas the absence of CD36 protects caveolae from cholesterol depletion and the translocation of eNOS out of caveolae and maintains the ability of acetylcholine to stimulate NO production. Caveolae-localized sphingomyelin may serve as the substrate for the generation of the ceramide that stimulates eNOS. In contrast to ox-LDL and CD36, which remove cholesterol from caveolae, HDL and SR-BI move cholesterol into them. This could serve as an indirect effect on eNOS function and helps to maintain the cholesterol level of caveolae, which allows eNOS to remain associated with caveolae [143].

Accumulating evidence exists that membrane lipid rafts and lipid platforms, respectively, may represent the important mechanisms by which redox signals are produced and transmitted in response to various agonists or stimuli [125]. In this regard, many studies have shown that lipid rafts or platforms may participate in the signaling of cell apoptosis associated with oxidative stress during activation of various death receptors. It has been well documented that death receptors, in particular, Fas and tumor necrosis factor receptor 1 (TNFR1), are localized in lipid rafts and that the receptors in lipid rafts can interact to stabilize the raft further and allow raft aggregation (i .e., clustering) and the recruitment of raftophilic molecules to the complex, producing massive signaling action. These lipid raft-derived platforms are also involved in the early alterations of cell functions during activation of death receptors, which could be physiological or pathological. It has been reported that various death factors bind to their receptors in individual lipid rafts and subsequently stimulate acid SMase to produce ceramide from sphingomyelin in endothelial cells. More recently, evidence is increasing that the formation of lipid raft-derived or ceramide-enriched membrane platforms may be altered by redox molecules. Reports indicate that SOD decreased, but O_2_˙¯ increased the formation of ceramide-enriched membrane platforms in the membrane of coronary arterial endothelial cells [122]. In other studies, H_2_O_2_ was also found to activate prosurvival signaling pathways, including activation of PI3 kinase/Akt and ERK1/2 by a lipid raft-dependent mechanism. In addition to this direct evidence, ROS were found to influence lipid-raft signaling or function through their actions on many lipid-raft components such as ceramide production, cholesterol, and related raft proteins [144, 145].

It has been indicated that induction of lipid oxidation through ROS can amplify foam cell formation through ox-LDL uptake and a subsequent clustering of ceramide-enriched lipid domains. In addition, ox-LDL may affect cell-surface turnover of ceramide-backbone sphingolipids and apoE-mediated uptake by LRP family members. This in turn leads to cell-surface expansion of ceramide-enriched domains and activation of apoE/LRP1/CD1-mediated antigen presentation. HDL-mediated lipid efflux, however, disrupts lipid membrane microdomains and prevents foam cell formation. It is concluded that lipid rafts and related oxidative processes play an important role in the formation of macrophage foam cells and thus in the progression of atherosclerosis [3].

In addition to the role of the lipid-raft redox-signaling network in alterations of macrophage behavior, this signaling network may also be importantly involved in cell deformability, thereby initiating or promoting atherogenesis. It has been indicated that disruption of lipid rafts by oxidants such as ox-LDL alters the cytoskeletal structure, including the extent of polymerization, stabilization, crosslinking, and membrane association. These molecular alterations may increase force generation by the cytoskeleton, resulting in a stiffening of the cytoskeleton and hence stiffening of the cell and plasma membrane. Increased force generation and increased stiffness may also elevate membrane tension and thereby influence the activity of various mechanosensitive ion channels. Direct evidence suggests that ox-LDL could disrupt lipid rafts, resulting in a series of pathological changes in the biomechanical properties of vascular endothelial cells and ultimately inducing endothelial dysfunction and atherogenesis [146].

Mitogen-activated protein kinases and receptor tyrosine kinases were first recognized as residing in caveolin-rich microdomains; certain GPCRs and associated molecules were subsequently shown to be enriched in these domains [147-150]. Besides these proteins, several other proteins are associated to or included in different rafts shown in Table **2** [151-156].

Little is known about how proteins localize to different lipid domains. Different mechanisms for lipid raft targeting have been proposed or described: (i) Proteins may bind to caveolin *via* a scaffolding domain located near the N-terminus of caveolin-1 and caveolin-3 [157, 158]. Many proteins that bind to caveolin contain a putative caveolin-binding motif (a loosely defined pattern of aromatic and nonaromatic residues) [159]. (ii) N-linked myristoylation (of a glycine residue following the initial methionine) or thioacylation with palmitate (palmitoylation of cysteine residues) causes partitioning into the lipid-ordered phase of lipid rafts [160-165]. Caveolins function not only as scaffolds that localize signaling proteins, but, in addition, can inhibit numerous enzymes, including AC, eNOS, and several kinases and serine/threonine phosphatases [166-173]. Consistent with the latter findings, data from studies with knockout animal models and from overexpression protocols suggest that caveolins play central roles in regulating signal transduction by various systems [173-177].

**Fig. (2):**
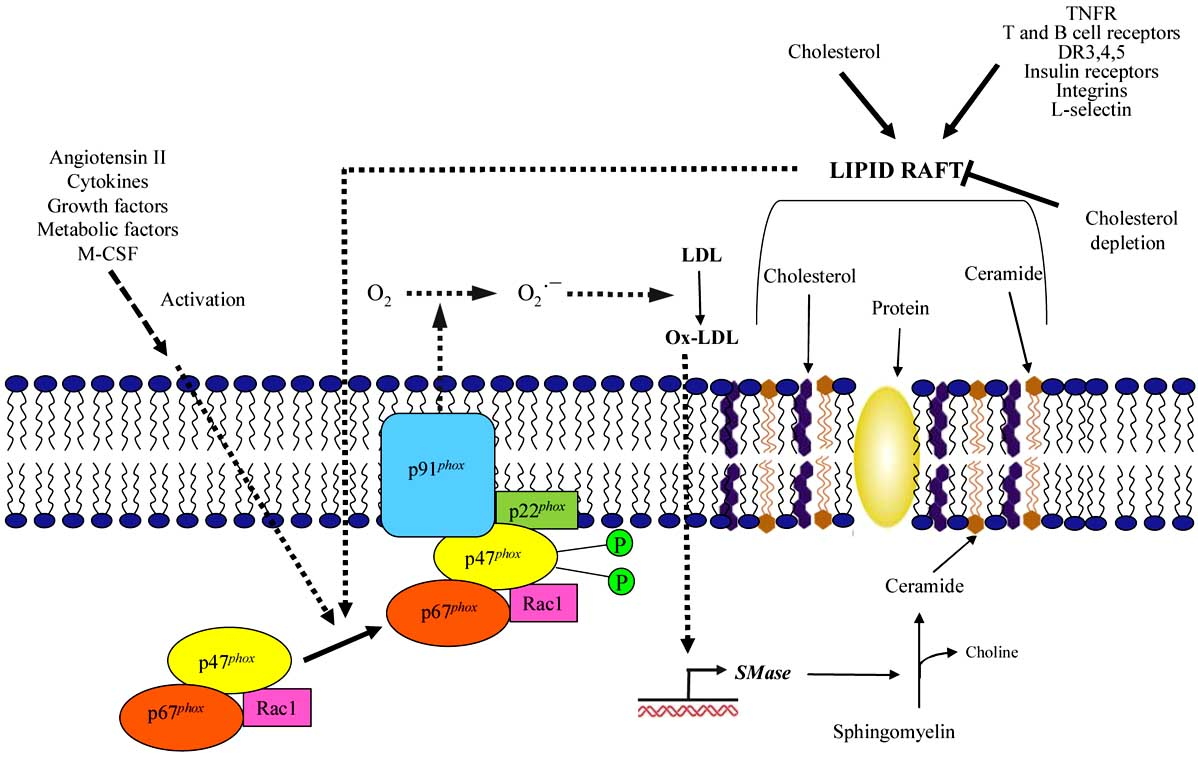
**NADPH oxidase and lipid raft signaling.** NADPH oxidase is activated by several factors, following the phosphorylation of p47*phox*, cytosolic subunits are bound to subunits in the membrane and then it catalyzes superoxide radical formation. Superoxide radical induces modification of LDL, expression of sphingomyelinase mRNA and degradation of sphingomyelin to ceramide. On the other hand, lipid rafts (cholesterol rich domains) are formed by several factors mainly by cholesterol. Receptors are aggregated in lipid raft clusters and these structures play important role in signaling mechanisms. Also NADPH oxidase is activated by lipid rafts. All these events increase oxidative stress in the cell.

M-CSF stimulates raft-associated NADPH oxidase, resulting in ROS formation, which regulates Akt and p38/MAPK, and thus contributes to monocyte/macrophage survival [178]. Superoxide-producing phagocyte NADPH-oxidase consists of a membrane-bound flavocytochrome b558 complex with the subunits gp91^*phox*^ and p22^*phox*^, and the cytosolic factors p47^*phox*^, p67^*phox*^, and the small GTPase Rac-1, which translocate to the membrane to assemble the active complex after cell activation. Activated Rac-1 stabilizes the NADPH oxidase complex and promotes the production of ROS, which is used for host defense as well as oxidation of LDL (Fig. **2**). ROS can also directly activate extracellular signal-regulated kinases (ERK), a member of mitogen-activated protein kinases (MAPKs), which regulate cell proliferation, differentiation, motility, and survival, and the PI3-kinase enzyme complex, creating a bridge between the MAPK and PI3-kinase pathways [179]. In addition, M-CSF can directly induce PI3-kinase activation and phosphatidylinositolphosphate formation, resulting in NADPH-oxidase-mediated ROS production, which leads to induced Erk activation and monocyte survival [180].

NADPH oxidase as well as the tyrosine kinases of the Src family (e.g., Lck, Fyn, and Lyn), which are lipid-modified signaling proteins, GPI-linked proteins, and adaptor proteins are constituents of raft-membrane microdomains. On receptor binding, immune receptors become raft associated, and additional components of the signaling pathways are recruited to rafts to form signaling complexes. The entry of immune receptors into rafts can regulate cell activation, and raft integrity is crucial for the initiation and maintenance of intracellular signals [181]. It has been shown that superoxide production by NADPH oxidase is inhibited by cholesterol depletion because of impaired translocation of cytosolic protein subunits to the membrane [182]. Formation of lipid rafts in the membrane of coronary endothelial cells induces clustering and activation of reduced NADPH oxidase, thereby forming a redox signaling platform on the cell membrane that mediates transmembrane signaling of death receptors, resulting in endothelial dysfunction [121]. However, the inappropriate or excessive action of the NADPH oxidase system results in chronic inflammatory disorders like atherosclerosis. The sphingolipid ceramide has been reported as one of the critical signaling molecules to mediate the activation of NADH/NADPH oxidase in different cells. Results demonstrated an induction of ceramide rafts on ox-LDL loading of human macrophages [183], which could be involved in the activation of NADH/NADPH oxidase.

## CONCLUSION

There is a large body of evidence connecting the effects of oxidative stress and related signaling mechanisms with atherogenesis. Redox signaling is shown to play crucial role in several cardiovascular diseases also in hypercholesterolemia induced atherosclerosis. Tightly regulated ROS production by a family of NADPH oxidases, which is especially important in redox signaling, are raft associated. Understanding how lipid raft associated redox signaling pathways promote the process of atherosclerosis can be a key factor for clinical approaches.

## Figures and Tables

**Table 1:** Scavenger Receptors Playing Role in the Cellular Uptake of Atherogenic Lipids and Lipoproteins [3]

Class	Members	Location	Function
A	Type I MSR-A Type II MSR-A Type III MSR-A MARCO	Cell membrane of macrophages Cell membrane of macrophages Cytoplasmic vesicles of macrophages	Uptake of Ox-LDL by macrophages, transformation of macrophages into foam cells
B	CD36 SR-BI	Monocytes/macrophages, platelets, endothelial cells, adipocytes Hepatocytes, steroidogenic cells, epithelial cells, macrophages	Uptake of Ox-LDL by macrophages, transformation of macrophages into foam cells, phagocytosis of apoptotic cells HDL receptor mediates the selective uptake of HDL cholesterol
C	SRCL	Endothelial cells from human umbilical vein and vascular endothelial cells of the heart	Binds galactose and fucose residues
D	CD68/macrosialin	Expressed on endolysosomal compartments and macrophages cell surfaces	Member of lysosomal-associated membrane protein, binds Ox-LDL *in vitro*, transformation of macrophages into foam cells
E	LOX-1	Expressed by vascular endothelial cells, macrophages in human and mice	Endocytic uptake and lysosomal degradation of Ox-LDL. Binding to Ox-LDL induces NFκB activation and inhibits MCP-1 upregulation in endothelial cells.
F	SREC	Expressed by vascular endothelial cells	
G	SR-PSOX PSR	Lipid-laden macrophages in human atherogenic lesions Macrophages, fibroblasts, epithelial cells	Recognizes phosphatidylserine and Ox-LDL Phagocytosis of apoptotic cells
Others	CD163	Macrophages	Receptor for hemoglobin–haptoglobin complexes and prevents macrophages from oxidative damage by decreasing heme/iron levels and ROS formation

MSR, macrophage scavenger receptor; MARCO, macrophage receptor with collagenous structure; SR-BI, Scavenger recptor class B; SRCL, scavenger receptor C-type lectin; SREC, scavenger receptor expressed by endothelial cell-I; SR-PSOX, scavenger receptor for phosphatidylserine and oxidized lipoprotein; PSR, phosphatidylserine receptor.

**Table 2:** Proteins Associated to or Included in Different Rafts and Their Functions Protein Function

Protein	Function
Glycosylphosphatidylinositol protein linked proteins	Several signaling mechanisms
Flotillins	Uptake of cholera toxin and endocytosis of glycosylphosphatidylinositol linked proteins
Src family kinases	Phosphorylation of proteins on tyrosine residues
Epidermal growth factor receptors	Binds ligands as epidermal growth factor and transforming growth factor α
Platelet derived growth factor receptors	Regulates cell proliferation, cellular differentiation, cell growth and development
Endothelin receptors	Calcium homeostasis
Phosphotyrosine phosphatase syp	Signal transduction, binds to activated platelet derived growth factor recept
Growth factor receptor bound protein 2	Signal transduction, proliferation of various cell types
MAP kinase	Gene expression, mitosis, differentiation, proliferation, and cell survival/apoptosis
Protein kinase C	Phosphorylation of hydroxyl groups of serine and threonine amino acid residues on the proteins and controlling their functions
